# First Antibiotic Prophylaxis Prescription Among Children With Sickle Cell Disease

**DOI:** 10.1001/jamapediatrics.2026.0653

**Published:** 2026-04-20

**Authors:** Jiajing Scarlette Shi, Erin LaFon, Ankit Sutaria, Brandon Kyle Attell, Mei Zhou, Amy Tang, Angela B. Snyder

**Affiliations:** 1 Georgia Health Policy Center, Andrew Young School of Policy Studies, Georgia State University, Atlanta; 2 Department of Public Management and Policy, Andrew Young School of Policy Studies, Georgia State University, Atlanta; 3 Jimmy and Rosalynn Carter School of Public Policy, Georgia Institute of Technology, Atlanta; 4 Georgia Department of Public Health, Atlanta; 5 Aflac Cancer and Blood Disorders Center, Children’s Healthcare of Atlanta, Atlanta, Georgia; 6 Emory University School of Medicine, Atlanta, Georgia

## Abstract

This cohort study compares antibiotic prophylaxis prescription timing among children with sickle cell disease born between 2008 and 2022 to identify whether suboptimal uptake of antibiotic prophylaxis is due to the timely receipt of the first prescription by 3 months of age.

Children with sickle cell disease (SCD) should begin antibiotic prophylaxis at age 2 to 3 months to prevent severe pneumococcal infections and related mortality, and they should continue through age 5 years.^1,2^ Daily antibiotic prophylaxis reduces infection incidence by 84% in this population.^3^ However, consistent use remains low, with fewer than 23% receiving more than 300 days of antibiotic prophylaxis within a year.^4,5^ Since 2006, newborn screening (NBS) programs have tested for SCD, providing families with SCD education and hematology referrals and prompting initiation of antibiotic prophylaxis by either the pediatrician or hematologist.^6^ This cohort study aims to identify whether suboptimal uptake of antibiotic prophylaxis is due to the timely receipt of the first prescription by 3 months of age.

## Methods

The Georgia State University institutional review board determined this study to be exempt because it was conducted as a secondary analysis of data collected for the public health surveillance of hemoglobinopathies under an active agreement between Georgia State University and the Georgia Department of Public Health. The study was conducted from January 2025 to December 2025. We constructed a population-level cohort of children with SCD born between 2008 and 2022 using Georgia NBS data. Prescription timing was obtained from the State Electronic Notifiable Disease Surveillance System (SENDSS) from birth to 12 months of age. Following the STROBE reporting guideline, we report hazard ratios (HRs), estimated differences in restricted mean survival time (RMST), and corresponding 95% CIs. *P* values were calculated using Wald tests from the Cox proportional hazards model, and all tests were 2 sided, with statistical significance defined as *P* < .05. Analyses were conducted using R, version 4.4.1.

We used the Kaplan-Meier method to estimate the cumulative probability of receiving the first antibiotic prophylaxis prescription by 3 months of age and applied a Cox proportional hazards regression model to identify factors associated with this outcome. Covariates included were urbanicity, sex, SCD genotype, and birth year.

Urbanicity was determined by applying Rural-Urban Commuting Area Codes to maternal addresses extracted from SENDSS. SCD genotype was categorized into the most severe form of SCD, sickle cell anemia (HbSS or HbS β^0^ thalassemia), and the other 2 milder forms. Birth year was included to account for potential temporal trends. Finally, we compared time to receiving the first antibiotic prophylaxis prescription between groups using RMST up to 3 months.

## Results

NBS identified 1951 children with SCD born in Georgia between 2008 and 2022. We excluded 238 cases due to insufficient geographic data, leaving 1713 children (867 female [50.6%]) in the final analysis cohort.

By 2 months of age, 1081 (63.1%) children had received their first antibiotic prophylaxis prescription (Figure). This probability increased to 81.8% (n = 1401) by 3 months and 98.9% (n = 1694) by 12 months. Children in rural areas had an 18% lower hazard of receiving prophylaxis by 3 months compared with their urban peers (HR, 0.82; 95% CI, 0.70-0.95; *P* = .01) (Table). The RMST at 3 months indicated that rural children received prophylaxis prescriptions 4.34 days later than urban children (HR, 4.34; 95% CI, 0.57-8.11; *P* = .02) (Table).

**Figure.**
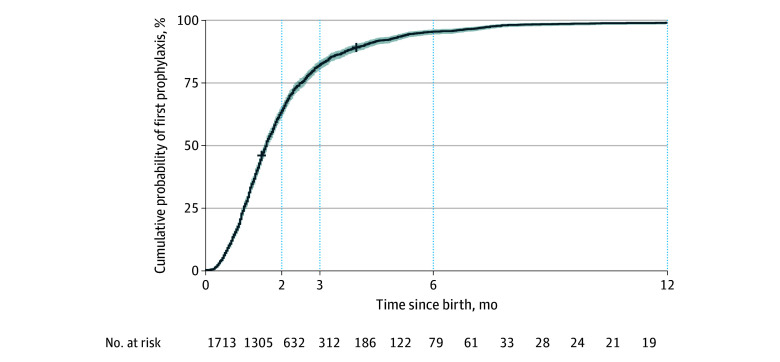
Kaplan-Meier Curve for First Prescription of Antibiotic Prophylaxis

**Table.** Demographic Cox Proportional Hazards Regression and Restricted Mean Survival Time Results by 3 Months of Age Among Children With Sickle Cell Disease

Characteristic	Children, No. (%)		Cox proportional hazards regression		Restricted mean survival time analysis^a^	
	Received antibiotic prophylaxis (n = 1408)	Did not receive antibiotic prophylaxis (n = 305)	HR (95% CI)	*P* value	Difference in days (95% CI)	*P* value
Sex						
Male	696 (49.4)	150 (49.2)	1 [Reference]		NA	NA
Female	712 (50.6)	155 (50.8)	0.96 (0.86-1.06)	.39		
Race^b^						
Black	1331 (94.5)	286 (93.8)	NA		NA	NA
Not Black	77 (5.5)	19 (6.2)				
Genotype						
Sickle cell anemia^c^	913 (64.8)	179 (58.7)	1 [Reference]		NA	NA
HbS β^+^ thalassemia	75 (5.3)	27 (8.9)	0.85 (0.69-1.06)	.16		
HbSC disease	420 (29.8)	99 (32.5)	0.95 (0.84-1.06)	.35		
Urbanicity						
Urban	1239 (88.0)	256 (83.9)	1 [Reference]		4.34 (0.57-8.11)	.02
Rural	169 (12.0)	49 (16.1)	0.82 (0.70-0.95)	.01		
Birth year						
2008-2010	266 (18.9)	56 (18.4)	1 [Reference]		NA	NA
2011-2013	268 (19.0)	66 (21.6)	0.87 (0.73-1.03)	.11		
2014-2016	286 (20.3)	67 (22.0)	0.95 (0.80-1.12)	.55		
2017-2019	298 (21.2)	59 (19.3)	1.04 (0.89-1.22)	.60		
2020-2022	290 (20.6)	57 (18.7)	0.97 (0.82-1.14)	.69		

Abbreviations: HR, hazard ratio; NA, not applicable.

^a^ Restricted mean survival time analysis was conducted only for urbanicity, as it was the only variable significantly associated with outcome in the Cox regression model.

^b^ Race was not included in the Cox regression model because most individuals with sickle cell disease in the cohort were Black.

^c^ Sickle cell anemia includes HbSS or HbS β^0^ thalassemia.

## Discussion

Using population data from Georgia, we found that more than 80% of children with SCD receive their first prescription for antibiotic prophylaxis by 3 months, with the probability increasing steadily to over 90% by 6 months. This suggests that Georgia’s NBS program is effectively facilitating evidence-based preventive care through timely prescribing of antibiotic prophylaxis. However, lower continued adherence to prescribed antibiotic therapy appears to be the major factor in reduced long-term compliance, as reported in previous studies.

Children in rural areas experienced a significant delay of approximately 4 days in receiving antibiotic prophylaxis prescriptions, which points to potential barriers related to access to SCD specialty care. Future public health efforts should increase adequate specialty care to rural communities, address transportation and resource limitations, and improve pediatrician education on SCD to reinforce compliance with antibiotic therapy and other preventive care. Study limitations include potential error in entering SENDSS information, unmeasured socioeconomic confounders affecting the outcomes, and the exclusion of children not captured in the data source and those with missing or incomplete records.

## Notes

Supplement.
Data Sharing Statement
