# Supplementary material for: First Antibiotic Prophylaxis Prescription Among Children With Sickle Cell Disease
Source: JAMA Pediatr. 2026 Apr 20;180(7):792–4. doi: 10.1001/jamapediatrics.2026.0653 (PMC13097030; doi:10.1001/jamapediatrics.2026.0653)
Supplement: Supplement. — Data Sharing Statement [file jamapediatr-e260653-s001.pdf]

Data Sharing Statement

Shi. First Antibiotic Prophylaxis Prescription Among Children With Sickle Cell Disease. *JAMA Pediatr*. Published April 20, 2026. doi:10.1001/jamapediatrics.2026.0653

Data
Data available: No

Additional Information
Explanation for why data not available: The data are housed by the Georgia Department of Public Health and are not publicly available.
